# Perspectives on Interval Exercise Interventions for Non-Alcoholic Fatty Liver Disease

**DOI:** 10.3390/medicines6030083

**Published:** 2019-08-01

**Authors:** Hidetaka Hamasaki

**Affiliations:** Hamasaki Clinic, 2-21-4 Nishida, Kagoshima, Kagoshima 890-0046, Japan; h-hamasaki@umin.ac.jp; Tel.: +81-099-250-3535; Fax: +81-099-250-1470

**Keywords:** exercise, high-intensity interval training, non-alcoholic fatty liver disease, type 2 diabetes, obesity

## Abstract

Non-alcoholic fatty liver disease (NAFLD) is the most common chronic liver disease and is associated with an increased risk of type 2 diabetes, cardiovascular disease, cirrhosis, and liver cancer. Exercise therapy is the most effective treatment for patients with NAFLD. High-intensity interval training (HIIT) is attracting attention as a time-efficient and an effective exercise modality for treating patients with NAFLD. Previous studies have shown that HIIT can reduce fat mass, visceral adipose tissue, and intrahepatic lipid levels and improve hepatic stiffness. HIIT may be an optimal exercise therapy to improve NAFLD in patients with a lack of time.

## 1. Introduction

Non-alcoholic fatty liver disease (NAFLD) is characterized by hepatic steatosis without viral infection, alcohol consumption, or any etiology of liver disease and is associated with an increased risk of type 2 diabetes (T2D), cardiovascular disease, cirrhosis, and hepatocellular carcinoma [1,2,3,4,5,6]. NAFLD is one of the most common causes of chronic liver diseases worldwide, particularly in patients with obesity, metabolic syndrome, and T2D [7]. Although there is currently no specific medicine for NAFLD, exercise plus diet appears to be the most effective intervention in managing patients with NAFLD [8]. Orci et al. [9] reported that exercise-based intervention, independent of dietary intake, reduced intrahepatic lipid levels in patients with NAFLD.

High-intensity interval training (HIIT) has recently been recognized as a novel exercise modality that is effective for the management of prediabetes [10], T2D [11,12,13], and overweight and obesity [14,15]; however, consensus regarding whether HIIT has benefits against NAFLD is lacking. HIIT is a time-efficient modality (e.g., 4 min of interval training separated by 3 min of recovery period × 4 sets); therefore, it may be an optional exercise strategy to improve NAFLD in patients with a lack of time.

## 2. Methods

The author searched the English literature of interval exercise and NAFLD using PubMed/MEDLINE. The search terms were “interval training/exercise” and “non-alcoholic fatty liver disease”. The search returned 61 published articles. The titles and abstracts of the identified articles were reviewed to determine their relevance. Studies were excluded if they were not randomized controlled trials.

## 3. Current Evidence 

Currently, four randomized trials have reported the effects of interval training in patients with NAFLD. Hallsworth et al. [16] reported that a 12-week HIIT, which involved cycle ergometry for 30–40 min thrice a week, significantly reduced intrahepatic lipid levels (−27%), fat mass (−1.8 kg), body fat percentage (−1.2%), and liver aminotransferase levels (-3 U/L). In addition, HIIT improved cardiac diastolic function in patients with NAFLD. Oh et al. [17] compared the effects of a 12-week HIIT, moderate-intensity continuous exercise training, and resistance training. HIIT comprised three sets of 3 min cycling at an intensity of 80–85% VO_2_max with 2 min rest at an intensity of 50% VO_2_max. Although intrahepatic lipid levels were similarly decreased in each group, an improvement in hepatic stiffness (−16.8%) was observed only with HIIT. Moreover, restored Kupffer cell phagocytic function was associated with this change in hepatic stiffness. Blood ferritin levels; expression of fat metabolism genes, such as sterol regulatory-binding protein-1c, acetyl-CoA carboxylase, and acyl-CoA oxidase; and blood leptin levels were also significantly reduced with HIIT. HIIT may improve hepatic fibrosis and inflammation with the pathophysiological change of NAFLD. In contrast, Winn et al. [18] reported no difference in intrahepatic lipid levels between exercise intensities. Exercise was performed on a treadmill for four weeks. Exercise energy expenditure was matched between HIIT and moderate-intensity continuous exercise training to evaluate the effects of exercise intensity alone on NAFLD. HIIT comprised 4 min exercise at an intensity of 80% VO_2_peak with 3 min recovery at 50% VO_2_peak, and continuous exercise training was performed at an intensity of 55% VO_2_peak; both exercise modalities reduced intrahepatic lipid levels (−37.0% and −20.1%, respectively) in patients with NAFLD. However, reduction in intrahepatic lipid levels did not significantly differ between the groups. Body weight, visceral abdominal tissue, liver aminotransferase levels, cytokeratin 18, and fetuin-A were not decreased in any group. The study duration might have been too short to result in a difference in hepatic steatosis between subjects who underwent energy-matched HIIT and those who underwent moderate-intensity continuous exercise training. Kamal et al. [19] investigated the effect of an 8-week HIIT program on intrahepatic triglyceride levels and health-related quality of life in diabetic patients with NAFLD. Subjects in the intervention group performed three sets of 4-min cycling at an intensity of 80%–85% VO_2_max with 2-min interval at an intensity of 50% VO_2_max thrice a week. The intrahepatic triglyceride levels, visceral adipose tissue, VO_2_peak, and health-related quality of life improved after the HIIT program. However, it is uncertain whether this HIIT program was effective in improving NAFLD because the study subjects were complicated with diabetes, and most of the subjects received metformin which may improve hepatic fat content [20].

Sprint interval training (SIT) is a variation of HIIT that can improve glycemic control and muscle mitochondrial capacity in patients with T2D [21]. SIT consists of brief (30 s) intervals of maximal-intensity exercise. Sargeant et al. [22] showed that a 6-week SIT program (4–6 maximal sprint cycling for 30 s thrice a week) significantly improved VO_2_peak and peripheral insulin sensitivity and reduced visceral adipose tissue (−16.9%) and intrahepatic triglyceride levels (−12.4%). MacLean et al. [23] showed that a 6-week SIT program (5–10 maximal sprint cycling for 6 s twice a week) did not reduce insulin resistance, liver aminotransferase levels, and the fibrosis-4 score in patients with NAFLD. However, both of these studies [22,23] were simple before–after studies without controls. Thus, the effects of the SIT program on NAFLD remain unknown.

## 4. Possible Physiological Mechanisms

The findings of animal studies [24,25,26,27,28] are highly suggestive of the physiological mechanism of the favorable impact of HIIT on NAFLD. Previous studies have shown that improved hepatic steatosis using HIIT is associated with increased hepatic mitochondrial function (citrate synthase activity and fatty acid oxidation), increased hepatic peroxisome proliferator-activated receptor (PPAR)-α content [24], increased hepatic PPAR-γ and glutathione peroxidase 4 gene expression [25], improved hepatic and adipose tissue insulin sensitivity independent of AMP-activated protein kinase phosphorylation of acetyl-CoA carboxylase [26], increased hepatic miR-122 expression [27], activated hepatic AMP-mediated protein kinase (AMPK) with upregulated adiponectin receptor 2 signaling pathway and downregulated NF-κB signaling pathway [28], and reduced hepatic M1 macrophage polarization markers [24], which suppress *de novo* hepatic lipogenesis [24,28]. AMPK-mediated improvement in mitochondrial function seems to play a key role in the treatment of NAFLD. In addition, exercise may improve NAFLD by enhancing autophagy [29]. Improved mitochondrial function via autophagy activation after HIIT may be associated with the reduction in hepatic steatosis in patients with NAFLD. Exercise reduces oxidative stress and hepatic gluconeogenesis [30], which is also a possible mechanism for the effect of HIIT on NAFLD.

## 5. Conclusions

Although the underlying mechanism remains unclear, HIIT can improve hepatic insulin sensitivity and reduce hepatic lipogenesis more effectively than conventional exercise in patients with NAFLD. However, some problems need to be solved. First, optimal HIIT program to effectively improve NAFLD has not been established. Second, patients who undergo HIIT must be supervised by training instructors because it is difficult for patients to keep following the exercise program by themselves. Patients with NAFLD may discontinue HIIT out of habit. Indeed, HIIT-induced improvements in patients with NAFLD are not sustained 12 months following the cessation of supervision [31]. Third, most study participants in previous studies were in their forties to fifties, and the beneficial effects of HIIT in older adults with NAFLD has not been verified. Clinicians should develop a safe, effective, and feasible HIIT program that patients with NAFLD, who are sometimes physically weak, can continue at home.
